# A Novel Pathogen of an Emerging Infectious Disease (Large Kidney Disease) in Farmed Blue Foxes

**DOI:** 10.1155/2023/6629054

**Published:** 2023-08-01

**Authors:** Yumao Wang, Chenchen Gu, Qiang Han, Shijun Fu, Jianjun Wang, Jinqiu Zhuang, Guangjun Guo, Jinfeng Liu, Xinyou Yu, Guanggang Qu, Zhiqiang Shen

**Affiliations:** ^1^Shandong Binzhou Animal Science and Veterinary Medicine Academy, Binzhou 256600, Shandong, China; ^2^College of Biology, China Agricultural University, Beijing 100019, China; ^3^Shandong Lvdu Biotechnology Co. Ltd, Binzhou 256600, Shandong, China; ^4^Binzhou Polytechnic, Binzhou 256600, Shandong, China

## Abstract

An emerging infectious disease (EID) in foxes called “large kidney disease”, characterized by enlarged kidneys, has been breaking out in fox farms in China, although its pathogenesis has not yet been elucidated. Here, we performed viral metagenomics sequencing on diseased fox tissue samples that identified a virus with 82.6% homology to the xenotropic murine leukemia virus-related virus (XMRV) PreXMRV-1 provirus strain (GenBank accession number NC_007815.2) in sick fox tissue. It was named PreXMRV-20, and its genome was verified by reverse transcription-polymerase chain reaction (PCR) and PCR product sequencing. Nonenveloped and polygonal virus-like particles consistent with the shape and size of XMRV were observed by negative staining electron microscopy. Administered subcutaneously, PreXMRV-20 infected weaned foxes, leading to growth retardation. The discovery of the PreXMRV-20 strain (the first isolation of an XMRV homolog in blue foxes) identifies a potential public health issue in blue fox breeding since XMRV has been confirmed to be a zoonotic virus.

## 1. Introduction

Blue foxes are genetically distinct coat color morphs of the Arctic fox *Vulpes lagopus*. Rare in nature, the blue fox has been successfully bred in captivity and is valued for its pelage. Over the years, however, a chronic and wasting disease of weaned foxes has emerged in fox farms across China, which is most common in blue foxes, although occasionally occurring in silver foxes and raccoon dogs. Characteristically, this emerging disease (EID) occurs in certain fox dens. The first sick foxes to show clinical symptoms were 10-day-old fox cubs, and the foxes after 15 days of weaning showed the most clinical symptoms. Symptoms mainly include gradual reduction in food intake, weight loss, retarded growth, messy hair of lighter color, the appearance of white hair, and swollen kidneys that can be palpated in the abdomen. The kidneys may be enlarged 2–3 times in diameter, hence the name “large kidney disease”. While the disease has been recognized for more than 20 years, the pathogenesis remains unclear [1, 2]. This EID is spreading in the Shandong province of China, and outbreaks are seen in increasing numbers of fox farms, with over 60% containing up to 10% sick animals. Sick foxes continue to die during the breeding process, and survivors are too small for fur pelts, which has caused great financial losses to farmers [3].

In this study, a virus with 82.6% homology to PreXMRV-1, a xenotropic murine leukemia virus (GenBank accession number NC_007815.2), was isolated and identified for the first time from a blue fox sick with large kidney disease in a fox farm in Shandong province of China. The discovery of this virus provides a direction for diagnosing and treating this EID.

## 2. Materials and Methods

### 2.1. Materials

#### 2.1.1. Laboratory Animals

Sick and dead blue foxes were from a farm in Shandong province, China. Healthy adult blue fox and eighteen 60-day-old weaned cubs were purchased from a local fox farm. Adult foxes and weaned cubs were housed in cages of different sizes. Every three weaned cubs were housed in one cage to reduce the stress response. Adult foxes are fed mainly carnivorous food, while weaned cubs need digestible feed.

#### 2.1.2. Main Reagents

DNA extraction kit (Beijing Century Yuanheng Animal Epidemic Prevention Technology Co., Ltd., Beijing); BL2000 PLUS DNA Marker (Beijing Boya Hongxing Technology Development Co., Ltd.); PrimeSTAR® HS DNA Polymerase (TaKaRa, Kusatsu, Japan), pMD19-T Vector (TaKaRa); DL2000 DNA Marker (TaKaRa); Gel Extraction Kit and Plasmid Mini Kit (Omega, Georgia, USA). (Omega). All other reagents used were analytically pure.

### 2.2. Methods

#### 2.2.1. Necropsy

Foxes were euthanized by intravenous pentobarbital sodium at a dose of 80 mg per kg to take organs. Fox organs were processed under strict biosafety conditions. Thin sections were stained by hematoxylin/eosin for histological examination following fixation and embedding in paraffin blocks.

#### 2.2.2. Tissue Preparation

Fresh tissue samples, including lung, kidney, liver, spleen, and brain samples, were removed. Kidney, liver, and brain samples were ground as 10% suspensions in PBS, freezethawed three times and clarified by low-speed centrifugation. Supernatants were diluted 1 : 9 in PBS, then shaken for 1 min with the same volume of chloroform. Following further clarification by low-speed centrifugation, the aqueous phase with added 1% penicillinstreptomycin (Gibco) filtrated by passage through a 0.22 *μ*m membrane and stored at −70°C for future use [4].

#### 2.2.3. Viral Metagenomic Sequencing

Ten milliliter filtrated mixture of fresh tissue samples, including liver, kidney, and brain tissue as Section 2.2.3 mentioned above, was sent to Shijiazhuang Boruidi Biotechnology Co., Ltd. for virus metagenomics sequencing analysis. The original data was obtained by Illumina Hiseq sequencing, and viral sequences were obtained by bioinformatics analysis of the original data.

#### 2.2.4. Phylogenetic Analysis

The gene fragments obtained by metagenomic sequencing were processed by MEGA 7 software [5] and were compared with published GenBank sequences for homology comparison and phylogenetic analysis.

#### 2.2.5. Primer Design and Synthesis

With the complete genomic sequence of the PreXMRV-1 provirus strain (GenBank accession number NC_007815.2) as a template, Primer 6.0 software was used to design two pairs of specific primers for XMRV detection toward the conserved region of the gag gene (Table 1). All primers were synthesized by Beijing TSING KE Biotechnology Co., Ltd.

#### 2.2.6. PCR of Pathological Materials

The cDNA transcribed from the pathological materials (Section 2.2.3) was used as a PCR template. PCR amplification was performed by XP1 and XP2 primers. The predicted length of the PCR product was separately 340 and 465 bp. The purified PCR product was inserted into the pMD19-T vector and used to transfect competent *Escherichia coli* DH5*α* cells. Following the screening on an LB plate containing ampicillin (100 *μ*g/mL), positive clones were sent to Sangon Biotech (Shanghai) Co., Ltd. for sequencing.

#### 2.2.7. Electron Microscope Observation

Ground kidney and lung suspensions were centrifuged at 5,000 g for 10 min to remove tissue fragments and examined by electron microscopy after negative staining with 0.5% phosphotungstic acid.

#### 2.2.8. Animal Experiment

Blood samples were collected from the 18 weaned cubs of blue foxes and subjected to reverse transcription-polymerase chain reaction (RT-PCR) to ensure all were PreXMRV-20 negative since the farm could only guarantee that the foxes were free from common diseases such as canine distemper and encephalitis. Eight were randomly selected and weighed. Six were injected subcutaneously (SC) with supernatant from the infected foxes, prepared as described above in Section 2.2.1. The remaining two were injected SC with PBS as controls. Animals were isolated separately for the duration of the experiment.

At 10 days postinjection, blood samples were taken for virus detection by RT-PCR. At 40 days, differences in growth rate between the control and experimental groups were apparent, and the cubs were reweighed. All were euthanized at 66 days as the experimental group had almost stopped eating, and organs were removed for pathological study. Brain samples were taken for virus detection by RT-PCR.

#### 2.2.9. Statistical Analysis

SPSS 17.0 software was used for statistical analysis. The experimental data were shown as mean value ± standard deviation. Statistical significance was defined as *P* < 0.01.

## 3. Results

### 3.1. Necropsy

**Figure 1:**
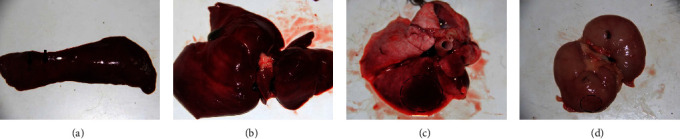
The necropsy of sick foxes. (a) Punctate hemorrhages in the spleen (black arrows). (b) The liver was swelling, hyperemic, and brittle. (c) Patchy hemorrhage in the lung (dashed area). (d) Renal cortex hemorrhage in the kidney (dashed area).

Pathological analysis of dead and sick foxes from a farm in Shandong province, China, revealed that foxes had enlarged spleens and punctate hemorrhages (Figure 1(a)). The liver was swelling, hyperemic, and brittle (Figure 1(b)). There was a patchy hemorrhage in the lung (Figure 1(c)). The kidney was pale, swelling, and stiff with renal cortex hemorrhage (Figure 1(d)).

### 3.2. Histological Examination of Pathological Tissues

**Figure 2:**
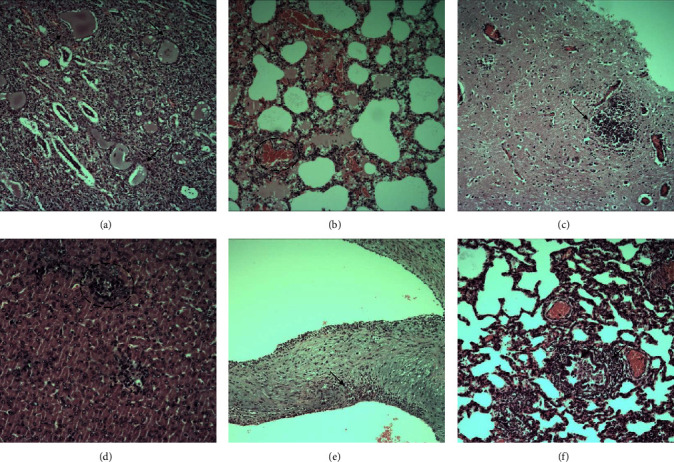
The histological observation of sick foxes. (a) Tubular inflammation (black arrows) in the kidney. (b) Alveolar hemorrhage (dashed area) and inflammatory cell infiltration (black arrows) in the lung. (c) Inflammatory cell infiltration (black arrow) in brain tissue. (d) Perivascular inflammation (dashed area) in the liver. (e) Inflammatory cell infiltration in endocardium (black arrow). (f) Peritracheal and endotracheal inflammation in bronchus (black arrows). Scale bar, 100 *μ*m.

Histologically, inflammatory cell infiltration was found in the kidney, lung, brain tissue, liver, endocardium, and bronchus (Figure 2). Additionally, calcium deposits were found in the kidney.

### 3.3. Viral Metagenomics Sequencing Result

DNA and RNA sequencing data confirmed the existence of a highly homologous virus of XMRV in tissues of diseased foxes. A highly homologous XMRV virus was isolated and identified from fox tissue samples for the first time.

#### 3.3.1. DNA Sequencing

An Illumina paired-end (PE) library was constructed by genome scanning through Illumina sequencing technology, and the sequencing data were then subjected to quality analysis (Figure S1), and low-quality data were removed before assembly (Table S1). Species taxonomy was determined by kraken2 (Table S2) [6]. Data were assembled by MEGAHIT [7]. Viral sequences were screened from the assemblies by comparison with the virus database, with results identifying a virus with 82.6% homology to the PreXMRV-1 provirus strain (Tables S3 and S4).

#### 3.3.2. RNA Sequencing

An Illumina PE library was constructed by genome scanning through Illumina sequencing technology, the sequencing data were subjected to quality analysis (Figure S2), and low-quality data were removed before assembly (Table S5). Matching reads were obtained by comparison with the Rfam database (Table S6). Species taxonomy was determined by kraken2 (Table S7). Data were assembled by MEGAHIT. Viral sequences were screened from the assemblies by comparison with the virus database (Tables S8 and S9).

### 3.4. Phylogenetic Analysis of PreXMRV-20

**Figure 3:**
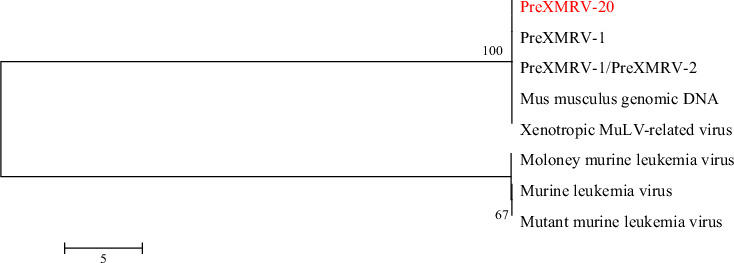
Evolutionary relationships among XMRV-related viruses. The tree with the highest log likelihood is shown. GenBank sources: PreXMRV-1: NC_007815.2; PreXMRV-1/PreXMRV-2: KF584051.1; *Mus musculus* genomic DNA: NC_000068.8; Xenotropic MuLV-related virus: JF274252.1; Moloney murine leukemia virus: NC_001501.1; Murine leukemia virus: AF019230.1; Mutant murine leukemia virus: EU075330.2.

**Figure 4:**
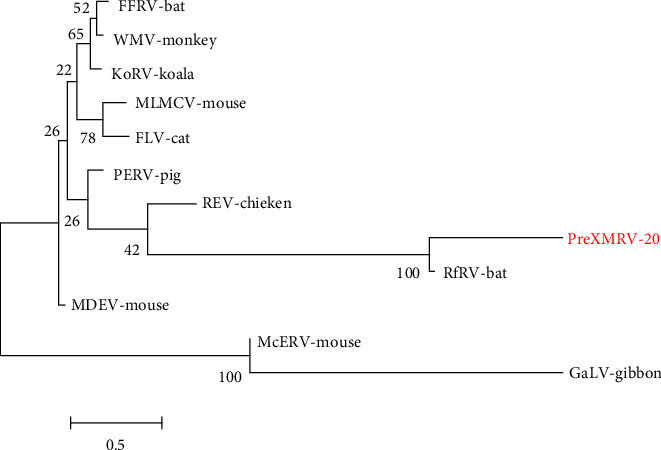
Molecular phylogenetic analysis of the gamma retroviral genome. The tree with the highest log likelihood is shown based on the general time reversible substitution model. GenBank sources: Reticuloendotheliosis virus REV: NC006934; *Rhinolophus ferrumequinum* retrovirus RfRV: JQ303225; Feline leukemia virus FLV: NC001940; Moloney murine leukemia virus MLMCV: NC001501; Gibbon ape leukemia virus GALV: NC001885; Woolly monkey virus WMV: Melomys wooly monkey virus MelWMV: KX059700; Koala retrovirus KoRV NC039228; *Mus caroli* endogenous virus McERV: KC460271; Mus dunni endogenous virus MDEV: AF053745; Porcine endogenous retrovirus PERV: AF038600; Flying-fox retrovirus FFRV: MK040728.

Sequence alignment of the gene fragments obtained by metagenomic sequencing of the unknown virus named PreXMRV-20 strain was done, and the evolutionary tree was constructed (Figure 3), which revealed that it shared the highest homology with PreXMRV-1, but also showed high homology with PreXMRV-2 and murine leukemia viruses (MLVs) indicating that PreXMRV-20 strain and XMRV may both be strains of MLV. An evolutionary tree constructed for gamma retroviruses shows that the PreXMRV-20 strain has the highest homology with bat-derived RfRV, suggesting that the virus may originate from bats (Figure 4).

### 3.5. Reverse transcription-PCR

**Figure 5:**
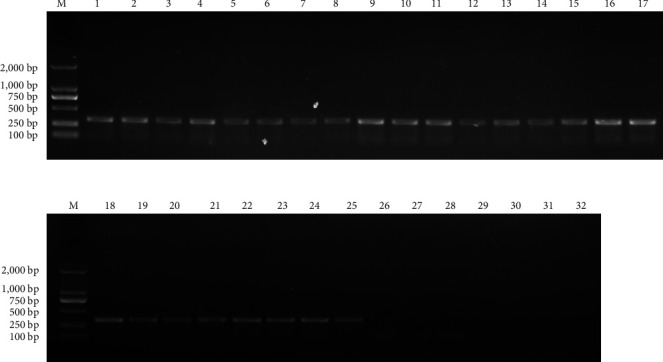
PCR amplification results of XP1 primers. M: DL2000 Marker. 1–5: the lung, spleen, kidney, brain, and liver of fox A. 6–10: the lung, spleen, kidney, brain, and liver of fox B. 11–15: the lung, spleen, kidney, brain, and liver of fox C. 18–20: the lung, spleen, kidney, brain, and liver of fox D. 21–25: the lung, spleen, kidney, brain, and liver of fox E. 26–30: the lung, spleen, kidney, brain, and liver of fox F. 31: blank control. 32: negative control.

**Figure 6:**
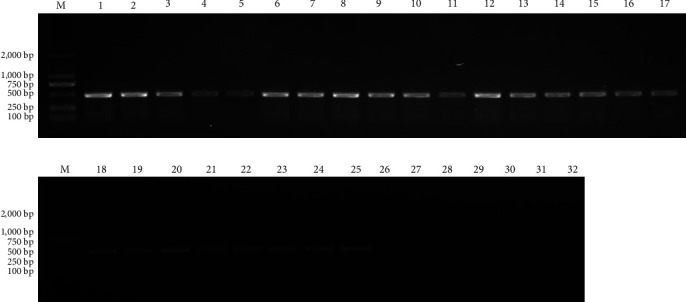
PCR amplification results of XP2 primers. M: DL2000 Marker. 1–5: the lung, spleen, kidney, brain, and liver of fox A. 6–10: the lung, spleen, kidney, brain, and liver of fox B. 11–15: the lung, spleen, kidney, brain, and liver of fox C. 18–20: the lung, spleen, kidney, brain, and liver of fox D. 21–25: the lung, spleen, kidney, brain, and liver of fox E. 26–30: the lung, spleen, kidney, brain, and liver of fox F. 31: blank control. 32: negative control.

Lung, liver, spleen, brain, and kidney samples were taken from five sick/dead foxes, and one healthy foxes (Table 2) after euthanasia, and RNA was extracted to obtain cDNA for use as a template, with two pairs of sequencing primers constructed as in 1.2.5, to perform PCR detection. All samples from foxes infected with PreXMRV-20 tested positive, showing the systemic distribution of the virus (Figures 5 and 6).

### 3.6. Electron Microscope Observation of PreXMRV-20

**Figure 7:**
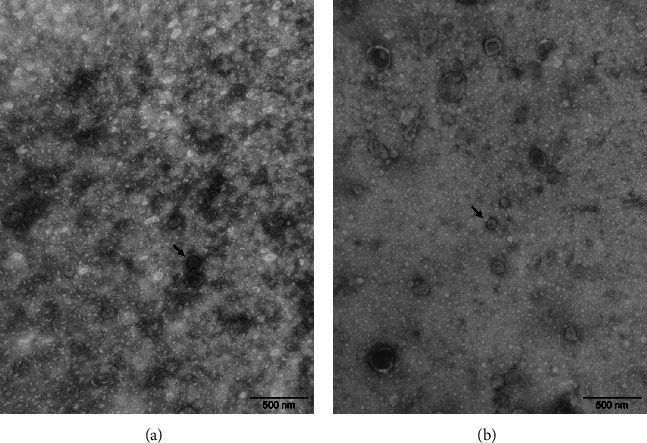
Morphology of viral particles under an electron microscope. (a) In the kidney, virus-like particles with no envelope and polygonal structure were observed (black arrow), and the shape and size were consistent with XMRV. (b) In the lung, virus-like particles with no envelope and polygonal structure were observed (black arrow), and the shape and size were consistent with XMRV.

The viruses in the kidney and lung tissues were observed by electron microscope (Figure 7). The viruses in the tissues were nonenveloped and polygonal, with a diameter of about 90 nm. The virus-like particles were consistent with the shape and size of XMRV.

### 3.7. The Results of the Animal Regression Experiment

#### 3.7.1. The Result of PreXMRV-20 Negative Foxes Screening

**Figure 8:**
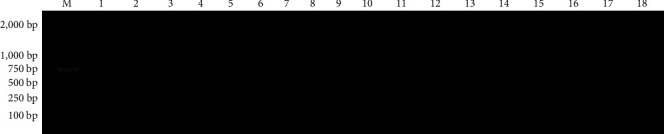
PCR amplification results of PreXMRV-20 negative foxes using XP1 primers. M: DL2000 Marker. 1–18: the weaned foxes.

The blood of 18 weaned foxes was taken to extract blood RNA, and cDNA was obtained by the reverse transcription. Using cDNA as the template, the XP1 primers constructed in method 1.2.5 were used for PCR detection. The result (Figure 8) showed that all 18 weaned foxes were PreXMRV-20 negative, six of which were randomly selected as the challenge group, and two of which were randomly selected as the control group.

#### 3.7.2. Postinjection RT-PCR Results

Six foxes in the challenge group were injected with the virus solution, and two foxes in the control group were injected with physiological saline by subcutaneous injection.

**Figure 9:**
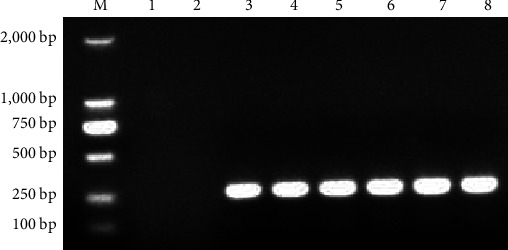
PCR amplification results of postinjected foxes using XP1 primers. M: DL2000 Marker. 1–2: the control group. 3–8: the challenge group.

On the 10th day after the challenge, the blood of the foxes in the challenge group and the control group was taken to extract the blood RNA, and cDNA was obtained by reverse transcription. cDNA was used as the template, and the XP1 primers constructed by method 1.2.5 were used for PCR detection. The result (Figure 9) showed that the control group was all negative for PreXMRV-20. The positive rate was 100%.

#### 3.7.3. Differences in Daily Weight Gain between Groups after Injection

The initial body weights of eight negative foxes were recorded before injection, and the body weights of the foxes in the experimental groups were measured 40 days after injection. The daily weight gain of the challenge group was 46.4 ± 0.3 g, while that of the control group was 58.6 ± 0.4 g. The daily weight gain of the challenge groups was significantly lower than that of the control group at 40 days (*P* < 0.01) (Table 3).

## 4. Discussion

Autopsy of sick and dead foxes revealed similar pathological changes in most organs as pallor, swelling, and sclerosis of the kidney with renal cortex hemorrhage. The farmers can only infer the type of disease based on the fox's incidence, clinical symptoms, and autopsy lesions. However, sending the materials of foxes to the laboratory for testing can only rule out some common pathogens such as canine distemper, canine parvovirus, Aleutian virus, and bacterial infection, and it is impossible to diagnose the pathogen without delay, let alone use targeted drug treatments. Therefore, there is yet to be an effective drug currently. The treatment is to eliminate or isolate the affected individuals mostly. It is also feasible to reduce the nutrition supply of the sick foxes to lower the burden of the kidneys and gradually increase the nutrient supply of the surviving foxes after the autumn equinox.

This study collected materials from blue foxes bearing “large kidney disease” for metagenomics sequencing. The sequencing results showed that there was a virus that was highly homologous to the PreXMRV-1 provirus strain in the diseased fox, named PreXMRV-20 strain. The results of animal regression experiments showed that PreXMRV-20 could infect weaned foxes and enter the brain tissue of diseased foxes. Necropsy of the cub showed the same tissue lesions as those found in foxes infected with “large kidney disease”, confirming that the virus is the infectious agent or can be used as a candidate pathogen. At present, the work of virus isolation and culture is in progress.

MLV, which belongs to the genus retrovirus, can induce cancer and other diseases in mice. According to the recipient's use, it is divided into biological, amphipathic, polyphilic, and xenogeneic groups [8].

XMRV belongs to a class of xenogeneic MLV. XMRV is the first *γ*-type retrovirus capable of infecting humans, which was first reported in 2006 and was discovered during the study of the pathogenesis of prostate cancer [9, 10], while its origin is unknown [11]. The PreXMRV-1 provirus is an endogenous retrovirus derived from XMRV, which can be passaged in human prostate cancer cells [12]. XMRV can infect human primary cells and cell lines, especially prostate-derived ones [13]. However, there is no report on whether other species can be infected.

Since XMRV is closely related to prostate cancer, research on XMRV is highly significant for predicting and diagnosing prostate cancer. According to reports, this virus may also be related to chronic fatigue syndrome (CFS) [14] and autism spectrum disorder (ASD) [15, 16]. Due to the relatively short time of XMRV discovery, the virus's infection rate in different populations and its relationship with the disease are not fully understood.

In this study, the PreXMRV-1 provirus-related virus PreXMRV-20 strain was found in foxes, the first report that this type of virus infects foxes at home and abroad. However, whether the PreXMRV-20 strain infects cells of mice and humans needs further verification. The results above provide a reference for the clinical diagnosis, prevention, and treatment of “large kidney disease” and lay a foundation for studying the pathogenesis. Since the PreXMRV-20 strain has the potential to infect humans, this discovery is of great significance to the protection of people who are in close contact with foxes and the prevention and control of the EID in public health.

## Figures and Tables

**Table 1:** Specific primers for XMRV detection.

Primer	Forward primer (5′–3′)	Reverse primer (5′–3′)
XP1	CCACTCAGTTGCCTAATGA	TCTTCTAACCGCTCTAACTT
XP2	CTCTCATTGACCTTCTCACA	TGACTTCATTAGGCAACTGA

**Table 2:** Sampling location and state of foxes.

Serial number	A	B	C	D	E	F
Age (days)	60	60	60	30	60	60

State	Morbid	Morbid	Deceased	Morbid	Deceased	Healthy

Sampling location	Lung	Lung	Lung	Lung	Lung	Lung
	Liver	Liver	Liver	Liver	Liver	Liver
	Spleen	Spleen	Spleen	Spleen	Spleen	Spleen
	Brain	Brain	Brain	Brain	Brain	Brain
	Kidney	Kidney	Kidney	Kidney	Kidney	Kidney

Samples were taken from sick foxes (A, B, and D), dead foxes (C and E), and healthy fox (F).

**Table 3:** Weight gains of cubs 40 days after injection.

Group	Serial number	Average daily gain (g)	Group daily gain (g)
The control group	1	59.25	58.63 ± 0.36
	2	58	

The challenge group	3	54.2	46.38 ± 0.33
	4	50	
	5	43	
	6	47.5	
	7	46.25	
	8	45.75	

Daily weight gain of the experimental groups was significantly lower than that of the control 40 days after infection (*P* < 0.01).

## Data Availability

The gene data used to support the findings of this study are included within the supplementary information file.

## Supplementary Materials

Supplementary Materials Figure S1: quality analysis of raw data of DNA sample. Figure S2: quality analysis of raw data of RNA sample. Table S1: sequencing data statistics. Table S2: notes on taxonomy of species. Table S3: assembly results. Table S4: Tthe comparison results. Table S5: sequencing data statistics. Table S6: rRNA ratio. Table S7: Notes on taxonomy of species. Table S8: assembly results. Table S9: the comparison results.
