# Supplementary material for: A Novel Pathogen of an Emerging Infectious Disease (Large Kidney Disease) in Farmed Blue Foxes
Source: Transbound Emerg Dis. 2023 Aug 1;2023:6629054. doi: 10.1155/2023/6629054 (PMC12017029; doi:10.1155/2023/6629054)
Supplement: Supplementary Materials — Figure S1: quality analysis of raw data of DNA sample. Figure S2: quality analysis of raw data of RNA sample. Table S1: sequencing data statistics. Table S2: notes on taxonomy of species. Table S3: assembly results. Table S4: Tthe comparison results. Table S5: sequencing data statistics. Table S6: rRNA ratio. Table S7: Notes on taxonomy of species. Table S8: assembly results. Table S9: the comparison results. [file 6629054.f1.docx]

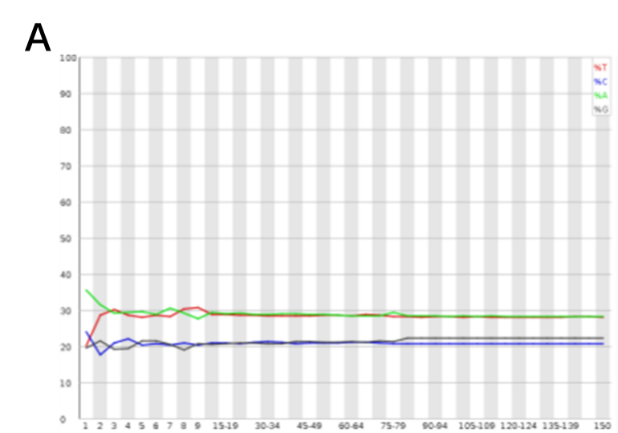

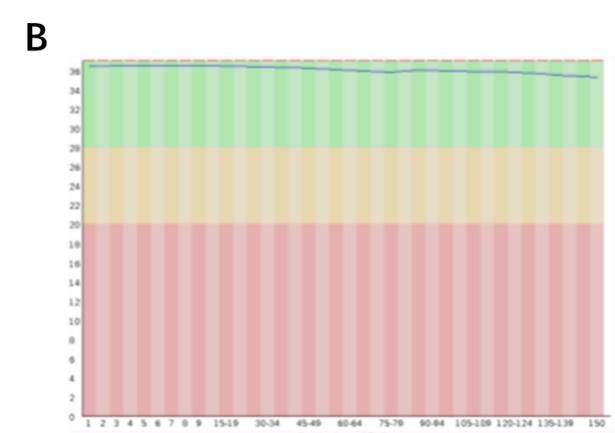


**Fig.S1 Quality analysis of raw data of DNA sample**

**(A)** Base composition of raw data. The horizontal coordinate is the reads base coordinate, and the vertical coordinate is the percentage of the A, C, G, T and N bases of all reads, respectively. At each position, A, C, G, and T fluctuate a little at the beginning, and then they stabilize. In general, A is equal to T and C is equal to G, and the percentage of each base will vary from species to species. In the case of uniform library construction, the dividing line of the four colors representing different bases should fluctuate very little and finally almost in a straight line. **(B)** Base quality of raw data. The horizontal coordinate is the reads base coordinate, and the vertical coordinate is the reads base quality. The range specified by the vertical red line in the figure is the combined quality of all reads bases. The red vertical square is the range of quartile values of the quality, and the bold black line is the median of the quality values.

**Table.S1 Sequencing data statistics**

| **Sample** | **Raw reads** | **Raw bases (bp)** | **Clean reads** | **Clean bases (bp)** |
| --- | --- | --- | --- | --- |
| HLA | 77678798 | 11651819700 | 73512416 | 10960085125 |

HLA: Sample ID; Raw reads: The number of raw sequence data reads; Raw bases: The number of original data series multiplied by the length; Clean reads: The number of reads of the sequence data after preprocessing; Clean bases: The number of sequences after preprocessing multiplied by the length.

**Table.S2 Notes on taxonomy of species**

| **ID** | **Eukaryota** | **Viruses** | **Archaea** | **Bacteria** | **Unclassify** | **Total Pair Reads** |
| --- | --- | --- | --- | --- | --- | --- |
| HLA | 1817907 | 7752 | 11873 | 213839 | 34704837 | 36756208 |

The preprocessed sequence data can be divided into Eukaryota, Viruses, Archaea, Bacteria, and Unclassify data five types after analysis by kraken2 software.

**Table.S3 Assembly results**

| **Sample** | **Ctg_num** | **Ctg_length_all** | **Ctg_n50** | **Ctg_n90** | **Ctg_max** | **Ctg_min** |
| --- | --- | --- | --- | --- | --- | --- |
| HLA | 1428838 | 858084544 | 632 | 371 | 16296 | 200 |

Data assembly was performed by MEGAHIT and thus screening viral sequences.

**Table.S4 The comparison results**

| **Ass.scaff.ID** | **Length** | **Virval.assesion.ID** | **Length** | **Identity(%)** | **Identify-**  **length** | **Species_anno** |
| --- | --- | --- | --- | --- | --- | --- |
| HLA__162803 | 609 | NC_009889.1 | 8406 | 75.067 | 373 | RD114 retrovirus, complete genome |
| HLA__296296 | 435 | NC_007815.2 | 8197 | 82.587 | 201 | PreXMRV-1 provirus, complete genome |
| HLA__544686 | 306 | NC_001702.1 | 8135 | 81.544 | 298 | Murine type C retrovirus, complete genome |

The alignment results were obtained by aligning the assembled sequences with a virus database. The results showed that there was a viral sequence with 82.587% homology to the heterotropic mouse leukemia virus-related virus (XMRV) PreXMRV-1 provirus strain.


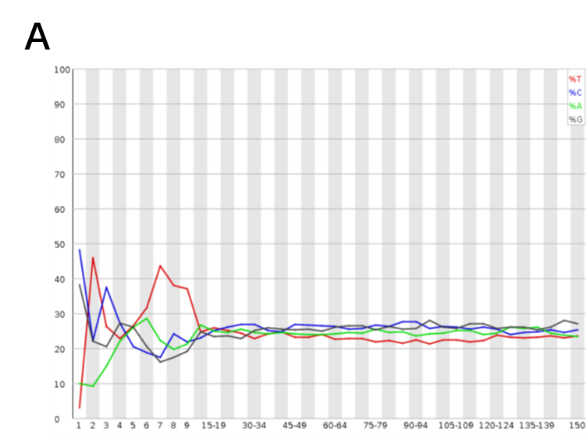

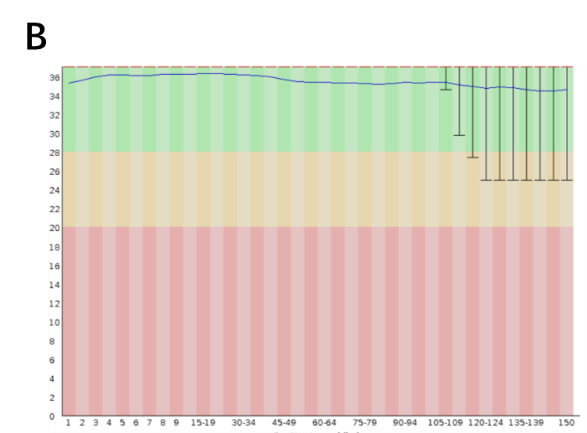


**Fig.S2 Quality analysis of raw data of RNA sample**

**(A)** Base composition of raw data. The horizontal coordinate is the reads base coordinate, and the vertical coordinate is the percentage of the A, C, G, T and N bases of all reads, respectively. At each position, A, C, G, and T fluctuate a little at the beginning, and then they stabilize. In general, A is equal to T and C is equal to G, and the percentage of each base will vary from species to species. In the case of uniform library construction, the dividing line of the four colors representing different bases should fluctuate very little and finally almost in a straight line. **(B)** Base quality of raw data. The horizontal coordinate is the reads base coordinate, and the vertical coordinate is the reads base quality. The range specified by the vertical red line in the figure is the combined quality of all reads bases. The red vertical square is the range of quartile values of the quality, and the bold black line is the median of the quality values.

**Table.S5 Sequencing data statistics**

| **Sample** | **Raw reads** | **Raw bases (bp)** | **Clean reads** | **Clean bases (bp)** |
| --- | --- | --- | --- | --- |
| HLA | 63136862 | 9470529300 | 53699356 | 8006896463 |

HLA: Sample ID; Raw reads: The number of raw sequence data reads; Raw bases: The number of original data series multiplied by the length; Clean reads: The number of reads of the sequence data after preprocessing; Clean bases: The number of sequences after preprocessing multiplied by the length.

**Table.S6 rRNA ratio**

| **Sample** | **Clean reads** | **rRNA reads** | **rRNA rate** |
| --- | --- | --- | --- |
| HLA | 53699356 | 727 | 0 |

Compared with the number of reads of the preprocessed sequence data, the number of rRNA reads was extremely low, and the rRNA ratio was 0, indicating that rRNA contamination was excluded.

**Table.S7 Notes on taxonomy of species**

| **ID** | **Eukaryota** | **Viruses** | **Archaea** | **Bacteria** | **Unclassify** | **Total Pair Reads** |
| --- | --- | --- | --- | --- | --- | --- |
| HLA | 371179 | 1533 | 2802 | 52566 | 26421598 | 26849678 |

The preprocessed sequence data can be divided into Eukaryota, Viruses, Archaea, Bacteria, and Unclassify five types after analysis by kraken2 software.

**Table.S8 Assembly results**

| **Sample** | **Ctg_num** | **Ctg_length_all** | **Ctg_n50** | **Ctg_n90** | **Ctg_max** | **Ctg_min** |
| --- | --- | --- | --- | --- | --- | --- |
| HLA | 240100 | 128054783 | 535 | 336 | 11337 | 200 |

Data assembly was performed by MEGAHIT and thus screening viral sequences.

**Table.S9 The comparison results**

| **Ass.scaff.ID** | **Length** | **Virval.assesion.ID** | **Length** | **Identify(%)** | **Identify-length** | **Species_anno** |
| --- | --- | --- | --- | --- | --- | --- |
| HLA__29976 | 452 | NC_001422.1 | 5386 | 97.768 | 448 | Coliphage phi-X174, complete genome |
| HLA__53292 | 2762 | NC_007815.2 | 8197 | 78.788 | 363 | PreXMRV-1 provirus, complete genome |
| HLA__53292 | 2762 | NC_001501.1 | 8332 | 79.027 | 329 | Moloney murine leukemia virus, complete genome |

The alignment results were obtained by aligning the assembled sequences with a virus database. The results showed that there was a viral sequence with 78.8% homology to the heterotropic mouse leukemia virus-related virus (XMRV) PreXMRV-1 provirus strain.
